# Acute Generalized Exanthematous Pustulosis in a Hemodialysis Patient

**DOI:** 10.7759/cureus.50354

**Published:** 2023-12-11

**Authors:** J'Moi Saunders Corea, Kaley B Schwartz, Ilya Fonarov, Damian Casadesus

**Affiliations:** 1 Internal Medicine, St. George's University School of Medicine, True Blue, GRD; 2 Primary Care, Orlando College of Osteopathic Medicine, Orlando, USA; 3 Hospital Medicine, Jackson Memorial Hospital, Miami, USA; 4 Internal Medicine, Jackson Memorial Hospital, Miami, USA

**Keywords:** hemodialysis, acetaminophen, vancomycin, pustular rash, drug reaction, staphylococcal scalded skin syndrome, acute generalized exanthematous pustulosis, dermatology, internal medicine

## Abstract

Acute generalized exanthematous pustulosis (AGEP) is an uncommon skin condition that should be considered when evaluating patients with severe skin eruptions accompanied by systemic symptoms. We present a woman in her 70s with end-stage renal disease on hemodialysis who developed a generalized pruritic rash seven days after the administration of pre-procedure vancomycin and acetaminophen. Our patient underwent a biopsy with findings consistent with AGEP. This report highlights the need to consider AGEP in patients with severe cutaneous eruptions and systemic involvement. Prompt biopsy and blood cultures are essential to prevent misdiagnosis and treatment delays.

## Introduction

Acute generalized exanthematous pustulosis (AGEP) is classified as a rare type IV hypersensitivity reaction of non-follicular pustules on an erythematous edematous base, occasionally involving mucosal sites. Although 90% of AGEP cases are drug-induced, some cases have described virologic and bacterial etiologies [1,2]. AGEP is common among multi-medicated patients and may range from localized and mild to severe, life-threatening reactions [1].

AGEP is often misdiagnosed in the clinical setting as Staphylococcal scalded skin syndrome (SSSS) and pustular psoriasis. Skin biopsy of these patients should be performed to confirm diagnosis due to their similar clinical presentation [2]. AGEP should be considered in patients with recent antibiotic administration and on multiple medications.

## Case presentation

A woman in her 70s with a prior history of hypertension, gout, congestive heart failure, atrial fibrillation, and end-stage renal disease on hemodialysis presented to the emergency department with a generalized rash. The rash was pruritic and diffuse, involving the face, chest, torso, back, bilateral upper extremities, and proximal bilateral lower extremities. The rash developed seven days after the administration of vancomycin for surgical prophylaxis of a left arm AV fistula placement. At the same time, the patient took acetaminophen for pain.

**Figure 1:**
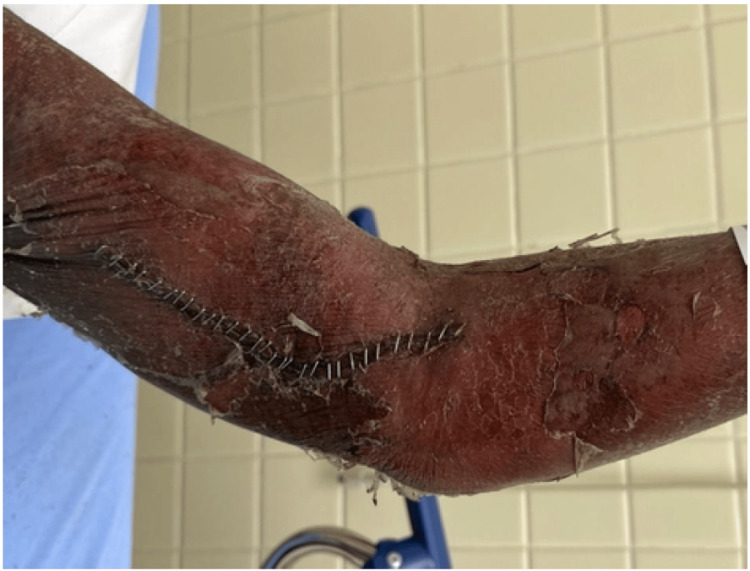
Left upper extremity with areas of exfoliating skin

**Figure 2:**
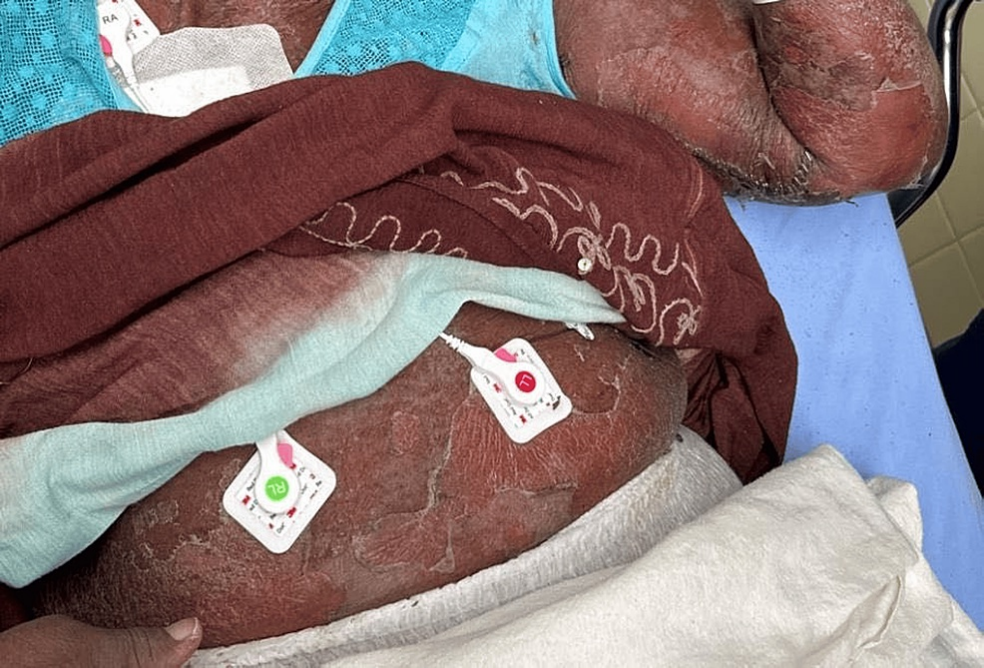
Diffuse fine pustules and areas of exfoliating skin sheets with superficial epidermal sloughing of the upper abdomen and left upper extremity

The patient's medications were allopurinol, atorvastatin, carvedilol, clopidogrel and sodium bicarbonate. No other medication was recently introduced. On the physical exam, the vital signs were a temperature of 36.6° C, heart rate of 68 beats/minute, respiratory rate of 18 breaths/minute, blood pressure of 93/42 mmHg, and oxygen saturation of 98% in ambient air. The respiratory, cardiovascular, and abdominal examinations were normal. Examination of the skin revealed diffuse fine pustules and areas of exfoliating skin sheets with superficial epidermal sloughing of the face, chest, torso, back, bilateral upper extremities, and proximal bilateral lower extremities (Figures 1-2). There was also oral mucosal involvement. Laboratory findings are presented in Table 1.

**Table 1:** Relevant initial laboratory values with reference values WBC - white blood count; BUN - blood urea nitrogen

Laboratory test	Patient laboratory value	Normal laboratory range
WBC	19.4 x 10^3^/mcL	3.8-10.8 x 10^3^/mcL
Hemoglobin	10.4 g/dL	11.7-15.5 g/dL
BUN	63 mg/dL	7-30 mg/dL
Creatinine	6.4 mg/dL	0.7-1.2 mg/dL

The patient was initially treated for presumed SSSS, given the skin findings, hypotension, and leukocytosis. Broad-spectrum antibiotics were administered, including linezolid 600 mg oral twice daily, cefepime 1 g intravenous (IV), and clindamycin 600 mg IV daily. Steroids were not administered due to the concern of infection. Vancomycin and acetaminophen were avoided since these were the possible offending agents. The patient underwent a right thigh skin biopsy. The biopsy revealed a sub-corneal collection of neutrophils with adjacent neutrophils, eosinophils, and rare dyskeratotic keratinocytes in the papillary dermis consistent with the diagnosis of acute generalized exanthematous pustulosis (AGEP).

## Discussion

**Figure 3:**
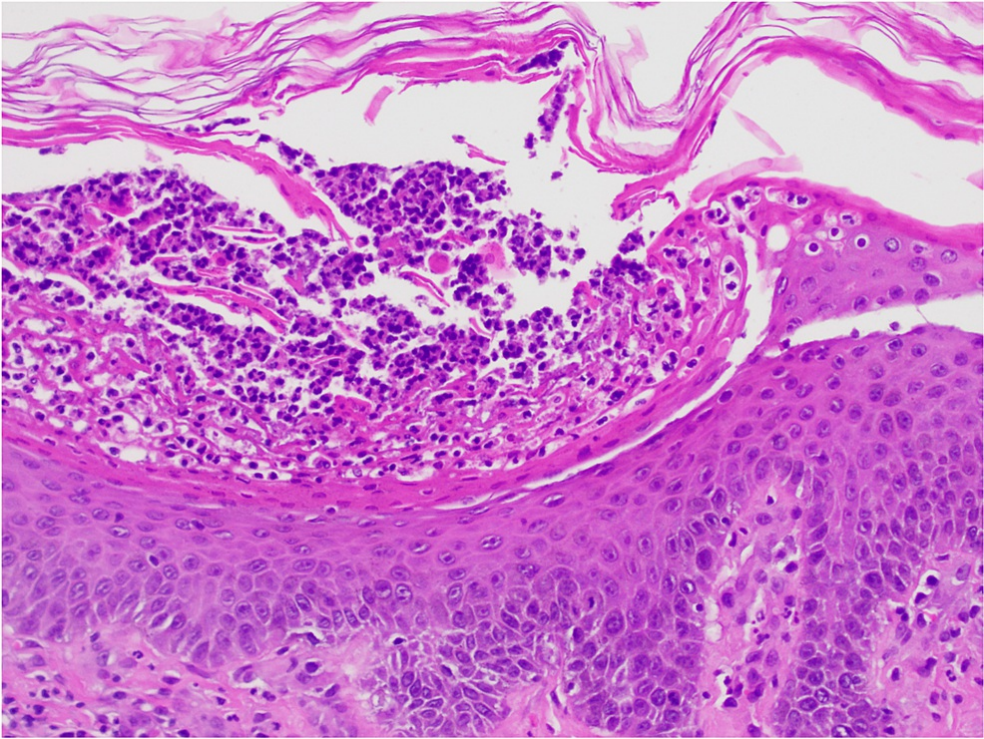
Skin with subcorneal pustule containing neutrophils Hematoxylin and eosin (20x)

**Figure 4:**
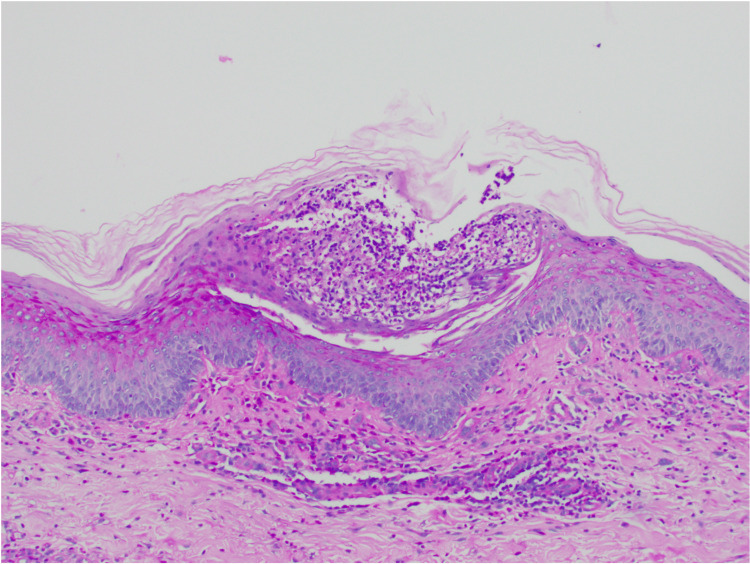
Negative PAS special stain for detecting fungal presence Periodic acid-Schiff (PAS; 4x)

The differential diagnosis upon presentation was pustular psoriasis, bullous impetigo, SSSS, and AGEP. Pustular psoriasis was not favored, given the presence of dermal eosinophils. Numerous reports have compared the similarities in clinical presentation between pustular psoriasis and AGEP, with many categorizing AGEP as a subdivision of pustular psoriasis [3]. Bullous impetigo was also possible, but bacterial forms were not identified in the biopsy (Figures 3-4). A periodic acid-Schiff stain was used to assess for the presence of fungi, and it was negative. SSSS was initially thought to be the diagnosis due to her septic presentation. There have been reported pediatric cases of SSSS coexisting with AGEP; however, there have been no clear reports in the elderly population [4]. The treatment of SSSS requires antibiotics, while the treatment of AGEP is supportive due to its self-limiting nature. The treatment of SSSS could exacerbate the symptoms of AGEP, as it is a drug-induced reaction.

It is unclear which medication precipitated this reaction. Vancomycin is implicated as the offending agent in three previous reports, and it could be the most likely causal agent in our patient [1,5]. Acetaminophen-induced AGEP has also been described in literature more commonly in the pediatric population [6]. Most recurrences are due to the administration of the same offending agent. However, there have been reports of relapsing AGEP with different agents, and it has been hypothesized to be a consequence of cytokine dysregulation [7].

AGEP is a rare adverse drug reaction induced by a type IV hypersensitivity T-cell mediated neutrophilic response causing erythematous-based sterile pustules [2]. While the disease course is usually self-limited, reports have shown that patients can be acutely at an increased risk of morbidity and systemic spread [1]. More severe cases warrant systemic corticosteroids; however, our patient was not administered steroids because of a presumed infection. Blood cultures were negative, and systemic antibiotics were discontinued. Literature has shown that there is no definitive treatment for AGEP despite the self-limiting course of the disease [1]. Once the diagnosis of AGEP was made in our patient, we recommended avoiding vancomycin and acetaminophen in the future. In addition, the patient was advised to use moisturizing agents and expect some post-inflammatory skin pigmentation. 

## Conclusions

AGEP is a rare cutaneous disorder and should be considered in the differential diagnosis of patients presenting with severe cutaneous eruptions with systemic involvement. Although we suspected that two agents had been implicated in causing AGEP in our patient, the specific offending agent was not identified. This highlights the limitations of identifying the causative agent, as they may be difficult to isolate. Most cases have shown to be drug-induced, and both acetaminophen and vancomycin were presumed to be the offending agents in this patient. Literature has reported cases of vancomycin-induced AGEP and acetaminophen-induced AGEP, although both are perceived to be uncommon causes of the disease. 
